# Supplementary material for: Follicular Immune Landscaping Reveals a Distinct Profile of FOXP3hiCD4hi T Cells in Treated Compared to Untreated HIV
Source: Vaccines (Basel). 2024 Aug 12;12(8):912. doi: 10.3390/vaccines12080912 (PMC11359267; doi:10.3390/vaccines12080912)
Supplement: Supplementary file 1 [file vaccines-12-00912-s001.zip › vaccines-3126402-supplementary.pdf]

Supplemental Table S1. Antibodies used for confocal imaging.

Antibody	*Clone*	*Dilution*	*Catalogue No*	*Imaging panel*
CD4	EPR6855	1/400	ab133616	1
Foxp3	236A/E7	1/100	ab20034	1
PD1	NAT105	1/100	3137	1
CD57	NK-1	1/200	Mob163	1
CD20	L26	1/400	NCL-L-CD20-L26	1
CD8	C8/144b	1/50	M7103	2
GrzB	GrB-7	1/40	MON7029C	2
CD68	PG-M1	1/150	M0876	2
CD163	10D6	1/200	NCL-L-CD163	2
CD15	MMA	1/1000	559045	2
CD16	2H7	1/100	CD16-L-CE	2

Supplemental Table S2. Mann–Whitney test-generated *p*-values were corrected using FDR correction with q = 0.05.

FIGURE 1D	*p*_value	*adjusted_p_value (FDR)*	FIGURE 1E	*p*_value	adjusted_*p*_value (FDR)	FIGURE 1G	*p*_value	*adjusted_p_value (FDR)*
1 VS 2	0.2222	*0.303*	1 VS 2	0.381	*0.5195455*	3 VS 4	0.1905	*0.1905*
1 VS 3	0.5368	*0.6193846*	1 VS 3	0.6255	*0.7217308*			
1 VS 4	0.0303	*0.04545*	1 VS 4	0.026	*0.065*			
1 VS 5	0.5368	*0.6193846*	1 VS 5	>0.9999	*0.9999*			
1 VS 6	0.005	*0.009375*	1 VS 6	0.0031	*0.016125*			
2 VS 3	0.0087	*0.0145*	2 VS 3	0.0043	*0.016125*			
2 VS 4	0.0043	*0.0092143*	2 VS 4	0.0043	*0.016125*			
2 VS 5	0.0043	*0.0092143*	2 VS 5	0.0519	*0.1112143*			
2 VS 6	0.0002	*0.001*	2 VS 6	0.0002	*0.003*			
3 VS 4	0.0022	*0.0066*	3 VS 4	0.1797	*0.26955*			
3 VS 5	0.8182	*0.8182*	3 VS 5	0.5887	*0.7217308*			
3 VS 6	<0.0001	*0.00075*	3 VS 6	0.0913	*0.1521667*			
4 VS 5	0.0022	*0.0066*	4 VS 5	0.0649	*0.1216875*			

4 VS 6	0.659	*0.7060714*	4 VS 6	0.968	*0.9999*			
5 VS 6	<0.0001	*0.00075*	5 VS 6	0.0064	*0.0192*			
FIGURE 2C	p_value	*adjusted_p_value (FDR)*	FIGURE 2D	p_value	*adjusted_p_value (FDR)*	FIGURE 2E	p_value	*adjusted_p_value (FDR)*
2 VS 3	0.9282	*0.9282*	2 VS 3	0.0035	*0.004375*	2 VS 3	0.0013	*0.0043333*
2 VS 4	0.7922	*0.8802222*	2 VS 4	<0.0001	*0.0002*	2 VS 4	<0.0001	*0.0005*
2 VS 5	0.2543	*0.4514286*	2 VS 5	0.1558	*0.1731111*	2 VS 5	0.0058	*0.0145*
2 VS 6	0.0034	*0.034*	2 VS 6	<0.0001	*0.0002*	2 VS 6	<0.0001	*0.0005*
3 VS 4	0.6767	*0.845875*	3 VS 4	0.0034	*0.004375*	3 VS 4	0.0168	*0.0336*
3 VS 5	0.3052	*0.4514286*	3 VS 5	0.4289	*0.4289*	3 VS 5	0.8635	*0.8635*
3 VS 6	0.0216	*0.072*	3 VS 6	<0.0001	*0.0002*	3 VS 6	0.1004	*0.1434286*
4 VS 5	0.316	*0.4514286*	4 VS 5	0.0014	*0.0023333*	4 VS 5	0.034	*0.0566667*
4 VS 6	0.0103	*0.0515*	4 VS 6	<0.0001	*0.0002*	4 VS 6	0.3476	*0.3862222*
5 VS 6	0.306	*0.4514286*	5 VS 6	<0.0001	*0.0002*	5 VS 6	0.118	*0.1475*
FIGURE 2F-1	p_value	*adjusted_p_value (FDR)*	FIGURE 2F-2	p_value	*adjusted_p_value (FDR)*	FIGURE 2G-1	p_value	*adjusted_p_value (FDR)*
1 VS 2	0.2874	*0.35925*	1 VS 2	0.8321	*0.8915357*	1 VS 2	0.393	*0.655*
1 VS 3	<0.0001	*0.0005*	1 VS 3	<0.0001	*0.000375*	1 VS 3	0.0041	*0.01025*
1 VS 4	0.0002	*0.00075*	1 VS 4	<0.0001	*0.000375*	1 VS 4	0.0029	*0.0087*
1 VS 5	0.0008	*0.0024*	1 VS 5	0.0072	*0.0173571*	1 VS 5	0.0555	*0.1040625*
1 VS 6	<0.0001	*0.0005*	1 VS 6	<0.0001	*0.000375*	1 VS 6	0.0014	*0.007*
2 VS 3	0.0013	*0.00325*	2 VS 3	<0.0001	*0.000375*	2 VS 3	0.0007	*0.007*
2 VS 4	0.0447	*0.0609546*	2 VS 4	0.0081	*0.0173571*	2 VS 4	0.0029	*0.0087*
2 VS 5	0.0359	*0.05385*	2 VS 5	0.1051	*0.1751667*	2 VS 5	0.0358	*0.0767143*

2 VS 6	<0.0001	*0.0005*	2 VS 6	0.0046	*0.0138*	2 VS 6	0.0012	*0.007*
3 VS 4	0.9241	*0.9547*	3 VS 4	>0.9999	*0.9999*	3 VS 4	0.5663	*0.74525*
3 VS 5	0.9547	*0.9547*	3 VS 5	0.0422	*0.079125*	3 VS 5	0.733	*0.8457692*
3 VS 6	0.0063	*0.0135*	3 VS 6	0.2106	*0.2871818*	3 VS 6	0.8773	*0.8773*
4 VS 5	0.913	*0.9547*	4 VS 5	0.1292	*0.1938*	4 VS 5	0.5096	*0.74525*
4 VS 6	0.0177	*0.0331875*	4 VS 6	0.4318	*0.53975*	4 VS 6	0.8763	*0.8773*
5 VS 6	0.0268	*0.0446667*	5 VS 6	0.4747	*0.5477308*	5 VS 6	0.5962	*0.74525*
FIGURE 2G-2	p_value	*adjusted_p_value (FDR)*	FIGURE 3D	p_value	*adjusted_p_value (FDR)*	FIGURE 3E	p_value	*adjusted_p_value (FDR)*
1 VS 2	0.5996	*0.6424286*	1 VS 2	0.0079	*0.05925*	1 VS 2	0.0079	*0.0535*
1 VS 3	<0.0001	*0.0005*	1 VS 3	0.329	*0.4813636*	1 VS 3	0.4286	*0.4945385*
1 VS 4	0.016	*0.0342857*	1 VS 4	0.0303	*0.113625*	1 VS 4	0.0823	*0.1543125*
1 VS 5	0.0015	*0.0045*	1 VS 5	0.1255	*0.2435625*	1 VS 5	0.0173	*0.064875*
1 VS 6	<0.0001	*0.0005*	1 VS 6	0.1299	*0.2435625*	1 VS 6	0.0107	*0.0535*
2 VS 3	0.0002	*0.00075*	2 VS 3	0.0043	*0.05925*	2 VS 3	0.0043	*0.0535*
2 VS 4	0.0633	*0.1055*	2 VS 4	0.329	*0.4813636*	2 VS 4	0.0519	*0.1112143*
2 VS 5	0.0104	*0.026*	2 VS 5	0.0303	*0.113625*	2 VS 5	0.2468	*0.3702*
2 VS 6	0.0001	*0.0005*	2 VS 6	0.0557	*0.16225*	2 VS 6	0.823	*0.8411*
3 VS 4	0.443	*0.5111539*	3 VS 4	0.0649	*0.16225*	3 VS 4	0.3095	*0.4220455*
3 VS 5	0.2066	*0.25825*	3 VS 5	0.4848	*0.5593846*	3 VS 5	0.0411	*0.10275*
3 VS 6	0.1827	*0.25825*	3 VS 6	0.7791	*0.83475*	3 VS 6	0.0408	*0.10275*
4 VS 5	>0.9999	*0.9999*	4 VS 5	0.3939	*0.492375*	4 VS 5	0.3939	*0.492375*
4 VS 6	0.202	*0.25825*	4 VS 6	0.353	*0.4813636*	4 VS 6	0.2074	*0.3456667*
5 VS 6	0.0346	*0.064875*	5 VS 6	0.8411	*0.8411*	5 VS 6	0.8411	*0.8411*

FIGURE 3H	p_value	*adjusted_p_value (FDR)*	FIGURE 4D-1	p_value	*adjusted_p_value (FDR)*	FIGURE 4D-2	p_value	*adjusted_p_value (FDR)*
5 VS 6	0.0007	*0.0007*	1 VS 2	0.3095	*0.661875*	1 VS 2	0.5476	*0.5867143*
			1 VS 3	0.0043	*0.0645*	1 VS 3	0.1255	*0.5288571*
			1 VS 4	0.6623	*0.827875*	1 VS 4	0.1255	*0.5288571*
			1 VS 5	0.6623	*0.827875*	1 VS 5	0.2468	*0.5288571*
			1 VS 6	0.2193	*0.54825*	1 VS 6	0.156	*0.5288571*
			2 VS 3	0.1775	*0.5325*	2 VS 3	0.2468	*0.5288571*
			2 VS 4	0.5368	*0.8052*	2 VS 4	0.2468	*0.5288571*
			2 VS 5	0.4286	*0.7143333*	2 VS 5	0.5368	*0.5867143*
			2 VS 6	0.8932	*0.9044*	2 VS 6	0.3913	*0.5371364*
			3 VS 4	0.132	*0.495*	3 VS 4	0.6991	*0.6991*
			3 VS 5	0.0087	*0.06525*	3 VS 5	0.3939	*0.5371364*
			3 VS 6	0.0757	*0.3785*	3 VS 6	0.1786	*0.5288571*
			4 VS 5	0.8182	*0.9044*	4 VS 5	0.3939	*0.5371364*
			4 VS 6	0.9044	*0.9044*	4 VS 6	0.353	*0.5371364*
			5 VS 6	0.353	*0.661875*	5 VS 6	0.494	*0.5867143*
FIGURE 4H	p_value	*adjusted_p_value (FDR)*	FIGURE 5C-1	p_value	*adjusted_p_value (FDR)*	FIGURE 5C-2	p_value	*adjusted_p_value (FDR)*
3 VS 4	0.4318	*0.6477*	1 VS 2	0.6905	*0.9999*	1 VS 2	0.6905	*0.8066538*
3 VS 5	0.0513	0.0931	1 VS 3	0.329	*0.5483333*	1 VS 3	0.5368	*0.8052*
3 VS 6	0.0431	0.0934	1 VS 4	>0.9999	*0.9999*	1 VS 4	0.6623	*0.8066538*
4 VS 5	0.4286	*0.6477*	1 VS 5	0.329	*0.5483333*	1 VS 5	0.2468	*0.5883333*
4 VS 6	0.6787	*0.7128*	1 VS 6	0.2193	*0.5483333*	1 VS 6	0.0143	*0.10725*

5 VS 6	0.7128	*0.7128*	2 VS 3	0.2468	*0.5483333*	2 VS 3	>0.9999	*0.9999*
			2 VS 4	0.7922	*0.9999*	2 VS 4	0.329	*0.5883333*
			2 VS 5	0.329	*0.5483333*	2 VS 5	0.329	*0.5883333*
			2 VS 6	0.1859	*0.5483333*	2 VS 6	0.087	*0.32625*
			3 VS 4	0.132	*0.5483333*	3 VS 4	0.3095	*0.5883333*
			3 VS 5	>0.9999	*0.9999*	3 VS 5	0.0649	*0.3245*
			3 VS 6	0.968	*0.9999*	3 VS 6	0.0046	*0.069*
			4 VS 5	0.1797	*0.5483333*	4 VS 5	0.6991	*0.8066538*
			4 VS 6	0.1297	*0.5483333*	4 VS 6	0.353	*0.5883333*
			5 VS 6	0.968	*0.9999*	5 VS 6	0.968	*0.9999*
FIGURE 5C-3	p_value	*adjusted_p_value (FDR)*	FIGURE 5C-4	p_value	*adjusted_p_value (FDR)*	FIGURE 5C-5	p_value	*adjusted_p_value (FDR)*
1 VS 2	0.6905	*0.873875*	1 VS 2	0.6905	*0.8698846*	1 VS 2	>0.9999	*0.9999*
1 VS 3	0.1255	*0.2689286*	1 VS 3	0.0519	*0.194625*	1 VS 3	0.1255	*0.585*
1 VS 4	0.9805	*0.9805*	1 VS 4	0.5368	*0.8052*	1 VS 4	0.6623	*0.9031364*
1 VS 5	0.0303	*0.10275*	1 VS 5	0.4286	*0.7143333*	1 VS 5	0.6623	*0.9031364*
1 VS 6	0.0194	*0.097*	1 VS 6	0.7539	*0.8698846*	1 VS 6	0.156	*0.585*
2 VS 3	0.1775	*0.2995*	2 VS 3	0.0823	*0.2469*	2 VS 3	0.2468	*0.617*
2 VS 4	0.7922	*0.9140769*	2 VS 4	0.329	*0.642375*	2 VS 4	0.9307	*0.9999*
2 VS 5	0.0087	*0.07725*	2 VS 5	0.329	*0.642375*	2 VS 5	>0.9999	*0.9999*
2 VS 6	0.0103	*0.07725*	2 VS 6	0.3426	*0.642375*	2 VS 6	0.2193	*0.617*
3 VS 4	0.1797	*0.2995*	3 VS 4	0.0022	*0.033*	3 VS 4	0.4848	*0.8233333*
3 VS 5	0.6991	*0.873875*	3 VS 5	0.0087	*0.06525*	3 VS 5	0.132	*0.585*
3 VS 6	0.968	*0.9805*	3 VS 6	0.0256	*0.128*	3 VS 6	0.494	*0.8233333*

4 VS 5	0.0411	*0.10275*	4 VS 5	0.9372	*0.9372*	4 VS 5	0.9372	*0.9999*
4 VS 6	0.0408	*0.10275*	4 VS 6	0.7181	*0.8698846*	4 VS 6	0.4442	*0.8233333*
5 VS 6	0.4442	*0.6663*	5 VS 6	0.9044	*0.9372*	5 VS 6	0.0913	*0.585*
FIGURE 5C-6	p_value	*adjusted_p_value (FDR)*	FIGURE 5E	p_value	*adjusted_p_value (FDR)*	SF3 A	p_value	*adjusted_p_value (FDR)*
1 VS 2	0.6349	*0.827875*	3 VS 4	0.2619	*0.2619*	1 VS 2	0.6571	*0.7581923*
1 VS 3	0.329	*0.827875*	3 VS 5	0.0667	*0.10005*	1 VS 3	0.3703	*0.692*
1 VS 4	0.6623	*0.827875*	3 VS 6	0.0312	*0.0936*	1 VS 4	0.6448	*0.7581923*
1 VS 5	>0.9999	*0.9999*	4 VS 5	0.0571	*0.10005*	1 VS 5	0.8055	*0.8055*
1 VS 6	0.3426	*0.827875*	4 VS 6	0.007	*0.042*	1 VS 6	0.0999	*0.692*
2 VS 3	0.3983	*0.827875*	5 VS 6	0.1419	*0.17028*	2 VS 3	0.4152	*0.692*
2 VS 4	0.9264	*0.9925714*				2 VS 4	0.5954	*0.7581923*
2 VS 5	0.5346	*0.827875*				2 VS 5	0.6167	*0.7581923*
2 VS 6	0.2968	*0.827875*				2 VS 6	0.1748	*0.692*
3 VS 4	0.132	*0.827875*				3 VS 4	0.3231	*0.692*
3 VS 5	0.5887	*0.827875*				3 VS 5	0.2829	*0.692*
3 VS 6	0.8411	*0.9705*				3 VS 6	0.4034	*0.692*
4 VS 5	0.4177	*0.827875*				4 VS 5	0.7617	*0.8055*
4 VS 6	0.0943	*0.827875*				4 VS 6	0.2677	*0.692*
5 VS 6	0.5059	*0.827875*				5 VS 6	0.1443	*0.692*
SF3C	p_value	*adjusted_p_value (FDR)*	SF4A-1	p_value	*adjusted_p_value (FDR)*	SF4A-2	p_value	*adjusted_p_value (FDR)*
2 VS 3	0.2595	0.0519	1 VS 2	0.6905	*0.7967308*	1 VS 2	>0.9999	*0.9999*
2 VS 4	0.827875	*0.827875*	1 VS 3	0.0173	*0.195*	1 VS 3	0.2468	*0.46275*

2 VS 5	0.2995	*0.3594*	1 VS 4	0.4286	*0.7967308*	1 VS 4	0.0823	*0.46275*
2 VS 6	0.2995	*0.3594*	1 VS 5	0.7922	*0.8487857*	1 VS 5	0.1775	*0.46275*
3 VS 4	0.2595	0.0411	1 VS 6	0.1859	*0.46475*	1 VS 6	0.1299	*0.46275*
3 VS 5	0.827875	0.5887	2 VS 3	0.0823	*0.4115*	2 VS 3	0.2468	*0.46275*
3 VS 6	0.9999	>0.9999	2 VS 4	>0.9999	*0.9999*	2 VS 4	0.0823	*0.46275*
4 VS 5	0.2995	*0.3594*	2 VS 5	0.6623	*0.7967308*	2 VS 5	0.2468	*0.46275*
4 VS 6	0.2995	*0.3594*	2 VS 6	0.5593	*0.7967308*	2 VS 6	0.2193	*0.46275*
5 VS 6	0.9817778	*0.8836*	3 VS 4	0.132	*0.4584*	3 VS 4	>0.9999	*0.9999*
			3 VS 5	0.026	*0.195*	3 VS 5	0.4848	*0.606*
			3 VS 6	0.1528	*0.4584*	3 VS 6	0.353	*0.5295*
			4 VS 5	0.4848	*0.7967308*	4 VS 5	0.4848	*0.606*
			4 VS 6	0.659	*0.7967308*	4 VS 6	0.3119	*0.5198333*
			5 VS 6	0.3119	*0.6683571*	5 VS 6	>0.9999	*0.9999*
SF5C-1	p_value	*adjusted_p_value (FDR)*	SF5C-2	p_value	*adjusted_p_value (FDR)*	SF5C-3	p_value	adjusted_p_value (FDR)
3 VS 4	0.64416	*0.5368*	3 VS 4	0.658	*0.329*	3 VS 4	0.7143	0.85716
3 VS 5	0.0909	*0.0303*	3 VS 5	0.658	*0.2468*	3 VS 5	0.1714	0.3428
3 VS 6	0.0012	*0.0002*	3 VS 6	0.658	*0.3132*	3 VS 6	0.0934	0.3428
4 VS 5	0.46425	*0.46425*	4 VS 5	0.8286	*0.8286*	4 VS 5	0.1143	0.3428
4 VS 6	0.329	*0.329*	4 VS 6	0.6594	*0.71205*	4 VS 6	0.2867	0.43005
5 VS 6	0.953	*0.953*	5 VS 6	0.9999	*0.9999*	5 VS 6	0.9451	0.9451
SF5D-1	p_value	*adjusted_p_value (FDR)*	SF5D-2	p_value	*adjusted_p_value (FDR)*			
3 VS 4	0.85536	*0.4286*	3 VS 4	0.51432	*0.4286*			

3 VS 5	0.85536	*0.5368*	3 VS 5	0.51432	*0.4286*			
3 VS 6	0.85536	*0.7128*	3 VS 6	0.2259	*0.0727*			
4 VS 5	0.9999	*0.9999*	4 VS 5	0.51432	*0.51816*			
4 VS 6	0.85536	*0.88752*	4 VS 6	0.2259	*0.2259*			
5 VS 6	0.85536	*0.88752*	5 VS 6	0.953	*0.953*			

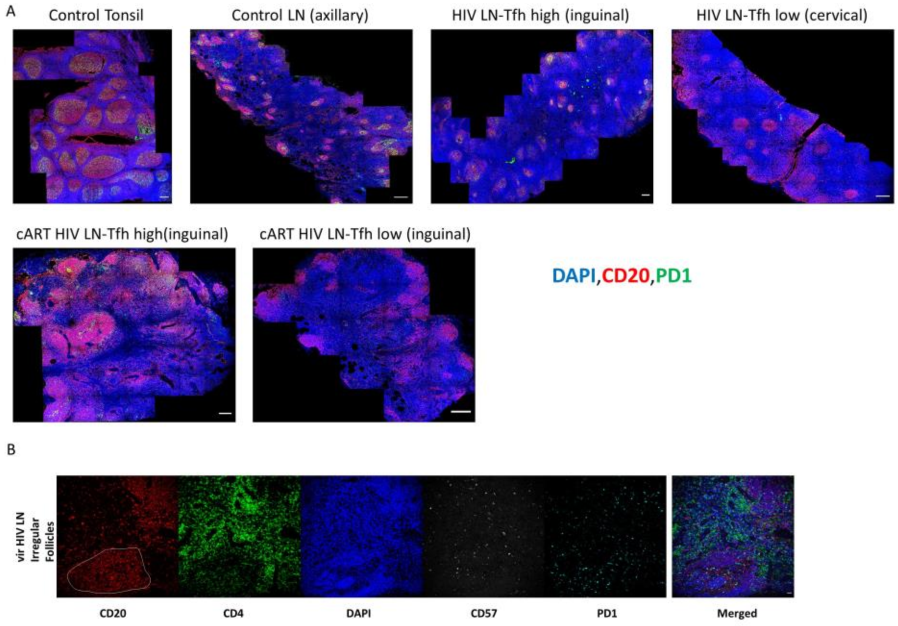

Supplemental Figure S1. (**A**) Representative whole-tissue examples of CD20 (red), PD1 (green) and DAPI (blue) staining pattern from control tonsils, control LNs, vir HIV LNs and cART HIV LNs (scale bar: 300 μm). (**B**) Representative examples of CD20 (red), CD4 (green), DAPI (blue), CD57 (gray) and PD1 (cyan) staining pattern from a cART HIV LN (scale bar: 30 μm). The white line denotes the follicular area.

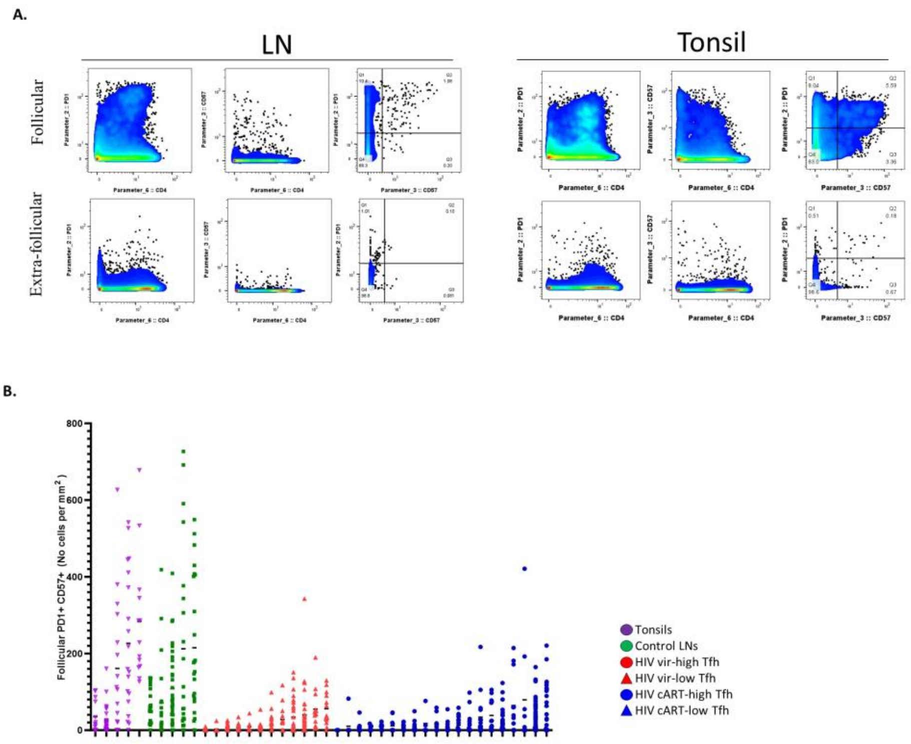

Supplemental Figure S2. (**A**) Immunophenotyping gating strategy used for the identification of T_{FH} cells in lymphoid and tonsillar tissues of interest, based on the expression of PD1 and CD57, by Histo-cytometry. The extrafollicular expression level was used for setting the gates identifying the PD1 and CD57 subsets. An example from one LN and one tonsil is shown. (**B**) Dot plot graph showing the normalized numbers of $PD1^{hi}CD57^{hi}$ T_{FH} cells in all groups. Each symbol represents a follicular area.

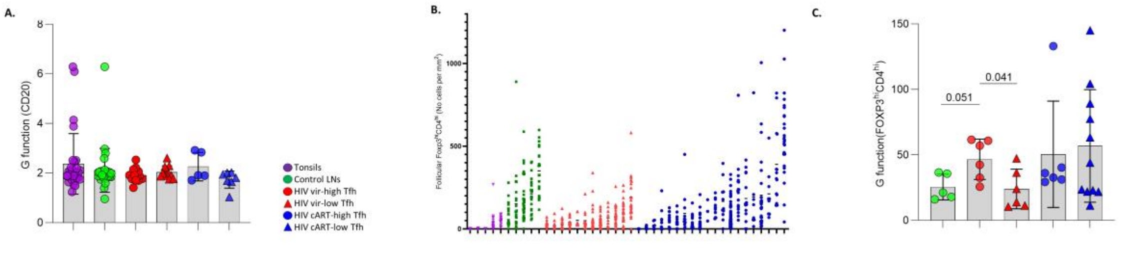

Supplemental Figure S3. (**A**) Bar graph showing the G function values for $CD20^{hi}$ B cells in all groups of tissues (control tonsils (N=29), control LNs (N=29), HIV vir-high T_{FH} (N=13), HIV vir-low T_{FH} (N=5), HIV cART-high T_{FH} (N=11) and HIV cART-low T_{FH} (N=7)). (**B**) Dot graph showing the distribution of normalized $FOXP3^{hi}$ $CD4^{hi}$ cell counts in tonsils, control LNs, vir HIV LNs and cART HIV LNs. Each symbol represents a follicle. (**C**) Bar graph showing the G function values for total $FOXP3^{hi}$ $CD4^{hi}$ T cells in control and HIV-infected LNs (control LNs (N=5), HIV vir-high T_{FH} (N=6), HIV vir-low T_{FH} (N=6), HIV cART-high T_{FH} (N=6) and HIV cART-low T_{FH} (N=11)). Each symbol represents a donor.

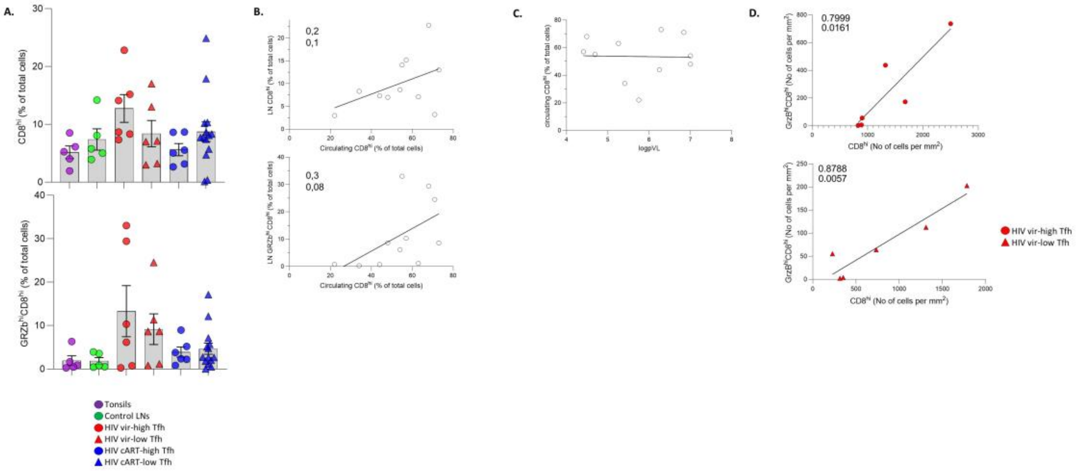

Supplemental Figure S4. (**A**) Bar graphs demonstrating the frequency of bulk CD8+ (upper panel) and GrzB^{hi} CD8+ (lower panel) cells in control tonsils (N=5), control LNs (N=5), HIV vir-high TFH (N=6), HIV vir-low TFH (N=6), HIV cART-high TFH (N=6) and HIV cART-low TFH (N=14). Each symbol represents a different donor. (**B**) Linear regression analysis between circulating CD8+ T cell frequencies and LN bulk or GrzB^{hi} CD8+ T cells in viremic PLWH. (**C**) Linear regression analysis between blood viral loads and frequencies of circulating CD8+ T cells in viremic PLWH. (**D**) Linear regression analysis between LN bulk and GrzB^{hi} CD8+ T cells in HIV viremic high- and low-TFH subgroups.

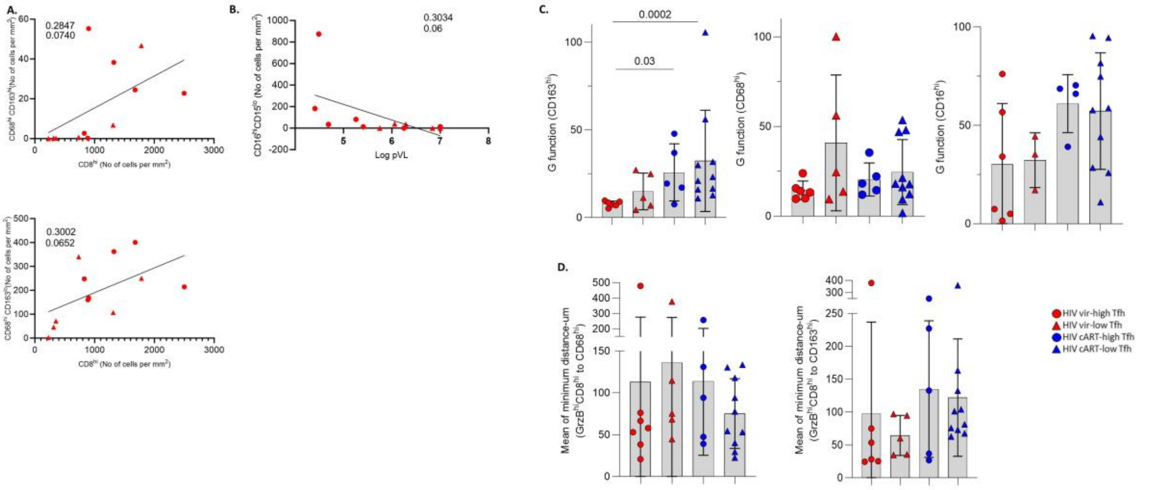

Supplemental Figure S5. (**A**) Linear regression analysis to show the correlation between LN CD68^{hi}CD163^{hi} (upper panel) or CD68^{hi}CD163^{lo} (lower panel) cells and LN CD8^{hi} cells in HIV vir LNs. (**B**) Linear regression analysis to show the correlation between LN CD16^{hi}CD15^{lo} and blood viral load in viremic PLWH. **(C)** Bar graphs showing the calculated G function values for CD163^{hi} (HIV vir-high TFH (N=6), HIV vir-low TFH (N=5), HIV cART-high TFH (N=5) and HIV cART-low TFH (N=10)), CD68^{hi} (HIV vir-high TFH (N=6), HIV vir-low TFH (N=5), HIV cART-high TFH (N=5) and HIV cART-low TFH (N=10)) and CD16^{hi} (HIV vir-high TFH (N=6), HIV vir-low TFH (N=3), HIV cART-high TFH (N=4) and HIV cART-low TFH (N=10)) LN cells in HIV subgroups. (**D**) Bar graphs showing the mean values of the minimum distances between GrzB^{hi} CD8^{hi} T cells and CD68^{hi} (left panel) or CD163^{hi} (right panel) cells in the HIV subgroups (HIV vir-high TFH (N=6), HIV vir-low TFH (N=5), HIV cART-high TFH (N=5) and HIV cART-low TFH (N=10)). Each symbol represents a different donor.

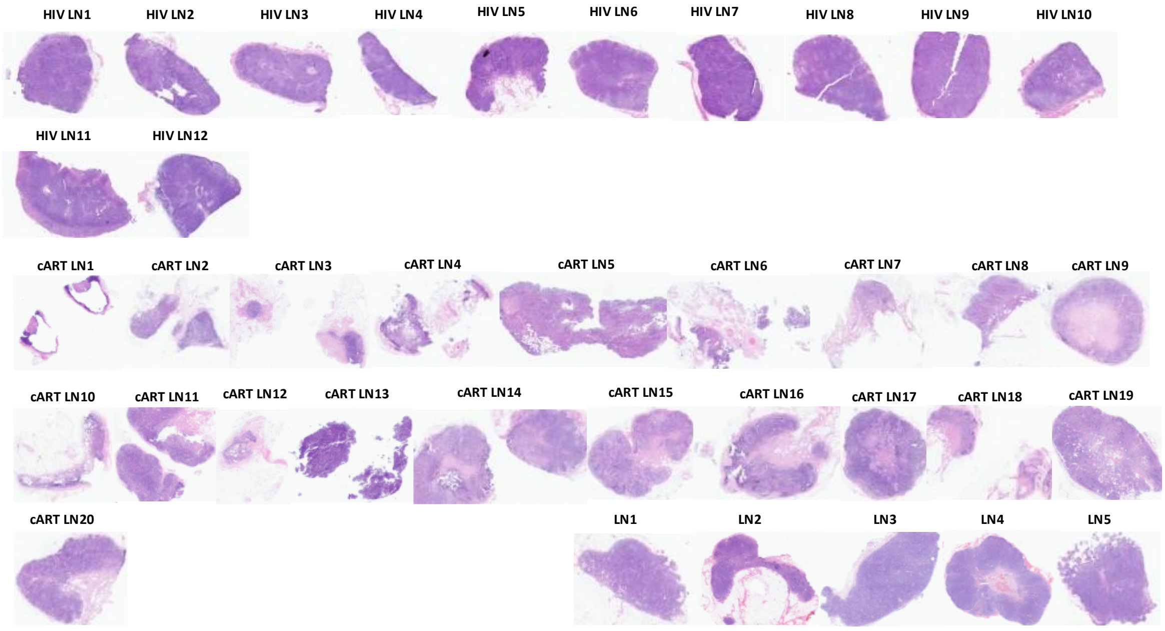

Supplemental Figure S6. Hematoxylin and eosin (H&E) staining for all the LN tissues used in this study (scale bar: 1.5 mm).
